# Preparation of Geopolymeric Materials from Industrial Kaolins, with Variable Kaolinite Content and Alkali Silicates Precursors

**DOI:** 10.3390/ma17081839

**Published:** 2024-04-16

**Authors:** Sergio Martínez-Martínez, Karima Bouguermouh, Nedjima Bouzidi, Laila Mahtout, Pedro J. Sánchez-Soto, Luis Pérez-Villarejo

**Affiliations:** 1 Department of Mechanical & Mining Engineering, Linares Higher Polytechnic School, University of Jaén, Cinturón Sur s/n, 23700 Linares, Spain; 2 Center for Advanced Studies in Earth Sciences, Energy and Environment (CEACTEMA), University of Jaén, Campus “Las Lagunillas”, 23071 Jaén, Spain; 3 Laboratoire de Technologie des Matériaux et de Génie des Procédés (LTMGP), Faculté de Technologie, Université A. Mira-Béjaïa, Terga Ouzemmour, Béjaïa 06000, Algeria; karima.bouguermouh@univ-bejaia.dz (K.B.); nedjima.bouzidi@univ-bejaia.dz (N.B.); laila.mahtout@univ-bejaia.dz (L.M.); 4 Institute of Materials Science of Sevilla (ICMS), Joint Center of the Spanish National Research Council (CSIC)-University of Sevilla, 41092 Sevilla, Spain; pedroji@icmse.csic.es; 5 Department of Chemical, Environmental and Materials Engineering, Linares Higher Polytechnic School, University of Jaén, Cinturón Sur s/n, 23700 Linares, Spain

**Keywords:** kaolinite, geopolymer, alkali silicates, sustainable foundations, sialate-siloxo type

## Abstract

In the present work, the development of geopolymeric materials with Na or K based on industrial kaolin samples, with variable kaolinite content and alkaline silicates, is studied. XRF, XRD, FTIR and SEM-EDS have been used as characterization techniques. Three ceramic kaolin samples, two from Algeria and one from Charente (France), have been considered. In particular, chemical and mineralogical characterization revealed elements distinct of Si and Al, and the content of pure kaolinite and secondary minerals. Metakaolinite was obtained by grinding and sieving raw kaolin at 80 μm and then by thermal activation at 750 °C for 1 h. This metakaolinite has been used as a base raw material to obtain geopolymers, using for this purpose different formulations of alkaline silicates with NaOH or KOH and variable Si/K molar ratios. The formation of geopolymeric materials by hydroxylation and polycondensation characterized with different Si/Al molar ratios, depending on the original metakaolinite content, has been demonstrated. Sodium carbonates have been detected by XRD and FTIR, and confirmed by SEM-EDS, in two of these geopolymer materials being products of NaOH carbonation.

## 1. Introduction

Geopolymeric materials or geopolymers are inorganic binders that began to be known in the 1970s as new materials with very interesting applications by J. Davidovits [1]. Originally, they referred to research resulting from the reaction of calcined kaolin (metakaolin) in a basic medium, using alkali or alkaline earth solutions, thus giving rise to the formation of a new class of aluminosilicate polymers that consolidated or set like a cement [1,2,3,4,5]. The prefix “geo” was chosen to symbolize their relationship to certain geological materials, such as natural stone or minerals. Today, “geopolymers” is considered a general term describing a wide variety of inorganic materials and composites, with no restrictions on their silica and alumina content, being defined as “low-temperature aluminosilicate glasses”, “hydroceramics”, “inorganic polymer cements” and even “alkali bonded ceramics” [6]. A definition of geopolymer materials has been proposed as essentially alkali-activated aluminosilicates, excluding any other alkali-activated materials and to be classified separately [5,6]. This is a “geosynthesis”, a reaction that integrates aluminosilicate-type minerals chemically; Si and Al react to form molecules that are chemically and structurally comparable to those that constitute natural rocks.

In a strongly alkaline solution, reactive aluminosilicate-type materials, such as metakaolin, dissolve rapidly and form hydroxylated oligomers of the Si(OH)^4−^ and Al(OH)^4−^ type [5,7]. During the polycondensation reaction, the tetrahedral units are alternately bonded together to form amorphous lattices that form geopolymers. In general, the properties of the materials obtained depend on the synthesis conditions, e.g., the choice of raw materials [8,9,10], Si to Al ratio [11,12], dilutions of alkali elements used, sodium or potassium [11,12,13] and addition of reinforcing materials [14]. All these variations in the composition of the obtained geopolymers are intended to improve their mechanical [15] or thermal properties [16,17]. However, it is important to note that the wide variety of possible geopolymer synthesis conditions results in the difficulty of determining whether the final material possesses a geopolymer lattice, despite the apparent full or partial consolidation of the geopolymer. The inorganic polymeric material could be considered as an amorphous material equivalent to feldspars (natural alkali aluminosilicates) but obtained by synthesis with the aid of a heat treatment and with a particular duration of the treatment, similar to certain organic polymers by polycondensation [1,2,3,4,5]. For example, previous studies on the synthesis of geopolymers from 2:1 silicates (kaolinite is of the 1:1 type) have shown that consolidation occurred, but no geopolymerization took place [18].

Geopolymers are very attractive materials from several points of view. We can highlight that they can be manufactured at room temperature, they have a relatively low cost since they are based on abundant and cheap raw materials for their manufacture, and materials that avoid the formation of temperature gradients (thermal stress) are obtained. They are dimensionally stable in a wide range of temperatures, they can be manufactured in situ and reinforcement materials (fibers, macromolecules, pigments, etc.) can also be added to obtain the required properties [5,6,9,10,11,12,13,14,15,16,17,19,20,21,22,23,24,25]. In addition, geopolymeric materials can also be reinforced by adding polymers in the production of hybrid materials [26,27,28,29,30,31]. These materials have been developed following the principles of “Green Chemistry”, as they have been obtained by synthesis from a wide variety of raw materials, including mineral by-products and recycled products, thus reducing energy demand and environmental impact during their production [19,20,25,32]. Geopolymers are the logical consequence of “Green Chemistry” in the service of sustainable development [6]. Geopolymerization can therefore be considered an environmentally sustainable technique, with promising developments for the coming years in uses such as the treatment of aluminosilicate-based waste, including the immobilization of toxic and radioactive materials [5,6,10,11,12,13,14,15,16,17,33,34,35,36,37,38,39,40,41,42]. Geopolymeric materials obtained with this technology can be applied in various fields, such as aeronautics and aerospace, foundry and non-ferrous metallurgy, construction, waste management, cultural heritage and many more [43,44,45,46,47,48,49,50,51,52,53,54,55,56,57,58,59,60,61,62,63,64,65,66,67,68]. They can also be applied in fields such as civil engineering, geotechnical engineering or mining engineering, as these materials can replace cement in the manufacture of foundations or, for example, in the stabilization of problematic clay soils for the paving of roads, bridges or tunnels, with advantages such as their ability to cement soils without the need for calcium, thus reducing CO_2_ emissions [69,70,71,72,73,74,75,76].

In addition to some applications already mentioned [12,13,14,15,16,17], the use of geopolymers as coatings on metallic substrates as thermal barriers [37], with an adhesion strength of more than 3.5 MPa on steel depending on their chemical composition, as well as their application as refractory adhesive material for metals and gaskets [40] and material resistant to chemical attack by acids [77], are of particular interest.

In recent decades, geopolymers have been seen as an environmentally viable alternative to Portland cement because of their performance. They have attracted attention for properties such as mechanical compressive strength, low permeability, good chemical resistance and durability against acid and sulfate attack, as well as excellent fire performance [5,13,15,33,34,35,36,37,38,40,77,78,79,80,81,82]. It has also been reported that obtaining geopolymers as alkali-activated inorganic materials reduces greenhouse gas (GHG) emissions [81,83]. These materials have been considered key to mitigating the carbon footprint and reducing the CO_2_ output of cements and concretes. It should be noted that the cement manufacturing industry is one of the largest producers of GHG emissions [84,85]. For example, CO_2_ contributes 65% to global warming, which must be taken into account in the current climate change conditions caused, among other reasons, by high CO_2_ emissions [78,80]. Geopolymer material hardens rapidly at room temperature, reaching its mechanical, chemical and thermal strength [3,4,5,12,13,15,17,34,35,36,37,38,40,86]. While cement owes its mechanical strength to the formation of hydrated calcium silicates, the exothermic geopolymerization reaction produces a structure similar to that formed in aluminosilicate gels and zeolites [2,10,37,38,39,86,87].

The preparation of geopolymers has been achieved from different raw materials among them, and kaolin is the most common [5,6,7,8,9,11,12,13,14,15,17,36,37,38,81,86,87,88], a raw material in which kaolinite is found in varying proportions [89], as well as clays [10,39], silica fume [16], fly ash [33,36,78], red mud from alumina production [87,90], slag and other industrial by-products and wastes [34,36,79,91], various sources of silica [16,77,92,93], coal ash [94] and others [36,95,96]. In essence, the kaolinite (1:1 aluminosilicate with structural OH groups) [89] found in kaolin, heat-treated until it loses structural OH and transforms into metakaolinite, is the main precursor, either as a single raw material or mixed with others, in the formation of geopolymeric materials [5,9,15,36,37,38,80,81,86,91,94,97]. According to the extensive literature on geopolymers, “metakaolin” (metakaolinite) is the raw material with the highest reactivity and purity compared to other raw materials, such as fly ash, red mud, slag, coal ash, kaolinitic clays and others already mentioned. The main problem with the other precursors is poor reproducibility due to a high variability of chemical compositions (red mud, fly ash, blast furnace slag, etc.). Therefore, the influence of the raw materials on the formation of geopolymeric materials is fundamental, especially with regard to the presence of impurities and the Si/Al molar ratio, as well as the reaction conditions, basic reagents used, their concentration, temperature and treatment time.

The use of 2:1 silicates in this type of synthesis [18,38,91] and the effect of the presence of other minerals of the kaolinite group, such as haloisite [89], can also be highlighted. In the latter case, it has been highlighted that this type of kaolin containing haloisite, once thermally treated, presents a higher rate of dissolution of Si and Al than the metakaolinite formed by the dehydroxylation of kaolinite, and this leads to better properties of the obtained geopolymer [9,95]. Likewise, the effect of the alkali cations present in the synthesis of geopolymers is also a prominent factor, as important modifications can be achieved in terms of the structure of the resulting geopolymeric materials, as, for example, has been demonstrated in a study on potassium-containing inorganic geopolymeric foams [96].

The aim of this work is to study the preparation of geopolymer materials from three industrial kaolins. These kaolins are first thermally treated for their activation and then chemically treated using basic solutions of alkaline silicates with different Na and K content. Both the solid raw materials and the geopolymer materials obtained are characterized by several techniques, mainly XRF, XRD, FTIR and the microstructures obtained by SEM-EDS. The effect of the composition of the reactive mixtures that have been formulated to obtain the geopolymer materials with different Si/Al molar ratios, kaolinite content that produces metakaolinite, as well as the influence exerted by the alkaline activator used (K or Na) are examined. The novelty of this work compared to the existing literature is the use of kaolin with different kaolinite content and the use of reactive alkaline solution varying the Si/K and Si/Na molar ratios.

## 2. Experimental

### 2.1. Solid Raw Materials: Kaolins

Three industrial kaolins were used for comparative purposes in this study. The first one is a kaolin from Charente (France) called K_CH. The other two kaolins studied come from Tamazert in El-Melia in Northern Algeria, called K_TA_1 and K_TA_2. The three kaolins were crushed and ground for homogenization once received in the laboratory, followed by sieving, obtaining the fraction below 80 μm which was used for this study.

### 2.2. Liquid Raw Materials: Alkaline Solutions

Two commercial sodium and potassium silicate solutions were used as chemical reagents, both supplied by Woellner (Wöllner GmbH, Ludwigshafen am Rhein, Germany), with Si/K and Si/Na molar ratios of 1.7 according to the manufacturer. The corresponding alkaline hydroxides were also used; specifically, KOH was supplied by Acros (Thermo Fisher Scientific Inc., Waltham, MA, USA) and NaOH was supplied by WVR (VWR International, LLC., Radnor, PA, USA), both in lentil form, with a purity of 85 and 99 wt%, respectively. By weighing and then carefully dissolving these hydroxides in the corresponding sodium and potassium silicates, strongly basic alkaline reagent solutions were obtained (pH close to 14) with similar molar ratios: Si/K = 0.58 and Si/Na = 0.56, taking into account the purity of the starting raw materials.

### 2.3. Preparation of the Geopolymeric Materials

The kaolins were treated at a temperature of 750 °C in air for 1 h in a laboratory furnace, using a porcelain capsule. In this way, the aim was to achieve a total dehydroxylation by heat treatment of the kaolinite contained in the raw materials and the formation of metakaolinite, which is much more reactive, to achieve the formation of geopolymer materials. Once the products of this heat treatment were cooled, they were mixed with the reactive alkaline solutions described in the previous subsection (Table 1). They were mixed vigorously and left to react for 20 min. The masses obtained were then placed in cylindrical, closable, alkali-resistant containers 15 mm in diameter and 30 mm high, and kept at 70 °C for 24 h.

The nomenclature used to designate the different synthesized geopolymers was G_K_TA_1_x, G_K_TA_2_x and G_K_CH_x, depending on the metakaolin used. In addition, the samples synthesized with K or Na are identified substituting x with K or Na at the end of the above designation.

The characterization of the materials obtained was carried out using different techniques, as described below.

### 2.4. Characterization Techniques

The chemical composition of the solid raw materials was determined by X-ray fluorescence (XRF), using a PANalytical Perl’X 3 unit (Malvern Panalytical, Malvern, UK), and pellets were prepared for this purpose by pressing. For the analysis of crystalline phases, X-ray diffraction (XRD) was used with a Bruker D8 (Bruker Corporation, Billerica, MA, USA), equipped with Debye Scherrer type instruments, CuKα radiation (1.5406 Å) and a graphite monochromator, scanning the area between 5° and 80° of 2θ at intervals of 0.02° and an acquisition time of 2 s.

The crystalline phases present in both the raw materials and the geopolymer materials obtained were identified by comparison with the standard Powder Diffraction Files (PDF) of the International Center for Diffraction Data (ICDD). In the case of quantitative analysis, the Rietveld method was used using NaF as the internal standard.

Analyses by Fourier Transform IR spectroscopy (FTIR) were carried out with a Shimadzu spectrophotometer model 8400 (Shimadzu Corporation, Kyoto, Japan), with a scanning range between 400 and 4000 cm^−1^. The samples to be examined were prepared by weighing and mixing the solid products (1 wt%) with KBr (99 wt%) and then pressing the obtained mixtures in the form of 10 mm diameter pellets.

The microstructure of the geopolymers formed was studied by scanning electron microscopy (SEM) with a JEOL SM 840 (JEOL Ltd., Tokyo, Japan) at 20 kV microscope, equipped with a chemical analyzer using Energy Dispersive X-ray Spectroscopy (EDS). The samples were placed in aluminum sample holders and coated with carbon using a JEOL JFC 1100 sputtering machine (JEOL Ltd., Tokyo, Japan).

The different characterization techniques, both of the raw materials and of the geopolymers obtained, have been carried out in triplicate, and an average was calculated to avoid possible errors.

## 3. Results and Discussion

### 3.1. Raw Material Characterization by XRF and XRD

The results of the chemical (XRF) and mineralogical (XRD) analysis of the three kaolins used for the preparation of the geopolymer materials are presented in Table 2 and Table 3.

As can be observed, Si and Al predominate, which is to be expected as these samples are considered as kaolins, although the K_CH sample presents the highest Al content of all of them (42.44 wt%), lower Si content and higher calcination loss at 1000 °C, which can be attributed to the other mineral phases present in addition to kaolinite. XRD mineralogical analysis of this sample confirms the presence of gibbsite (13 wt%), aluminum hydroxide, in this kaolin. Therefore, its presence leads to an increase in the percentage of Al determined by XRF and also in the weight loss by heat treatment as more structural OH groups are removed and not only from the kaolinite. The weight losses by heat treatment at 1000 °C are similar for the two Algerian kaolins K_TA_1 and K_TA_2 (10.50 wt%) and different for kaolin K_CH (16.00 wt%), as it contains this other hydroxylated mineral phase that contributes to the observed loss.

In general, in the three kaolins studied, the content of impurities other than Si and Al is variable, with minimum relative contents of Ca, Mg and Na, but high relative contents of K in samples K_TA_1 and K_TA_2. This is associated with the presence of muscovite (16–13 wt% as determined by XRD). The presence of a small relative amount of goethite is also observed in the Algerian kaolins. Overall, these are important differences with the K_CH kaolin, which shows the minimal contents of all these elements. The kaolinite content of these raw materials, as determined by XRD, is variable and is found to be 57 wt%, 66 wt% and 85 wt% by weight for the K_TA_1, K_TA_2 and K_CH kaolins, respectively.

**Figure 1:**
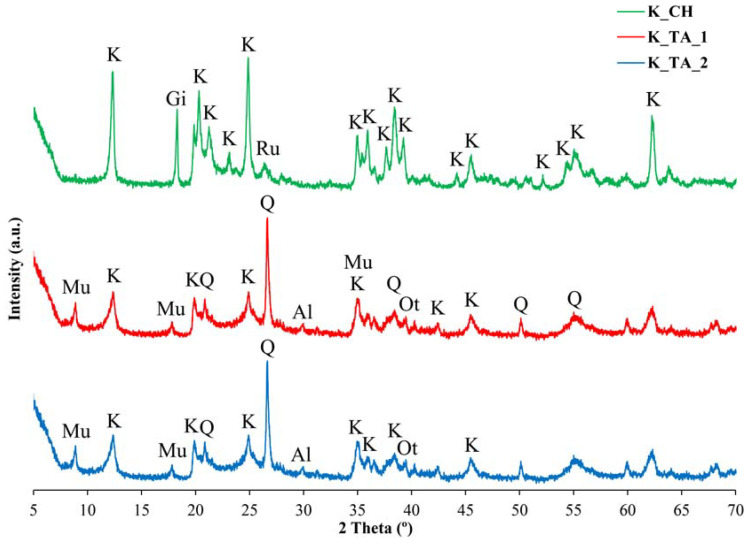
XRD of the kaolins used as raw material and treated at 750 °C for 1 h to form metakaolinite: kaolin K_CH (green line), kaolin K_TA_1 (red line), kaolin K_TA_2 (blue line). Symbols: K = Kaolinite, Q = Quartz, Mu = Muscovite Mica, Al = Albite, Ot = Orthose Feldspar, Gi = Gibsite, Ru = Rutile.

Figure 1 shows the XRD diffractograms of the three kaolins before they were heat-treated to be transformed into metakaolins, and then alkaline activated by either the potassium-based or the sodium-based activator. As can be seen, kaolin K_TA_1 (Figure 1—red line) is mainly composed of kaolinite of acceptable crystallinity, accompanied by quartz and muscovite mica, as well as feldspars (albite and orthoclase). Kaolin K_TA_2 (Figure 1—blue line) is richer in kaolinite than K_TA_1, with a lower relative content of the rest of the mineral phases. The diffractogram of kaolin K_CH (Figure 1—green line) shows that this kaolin has a higher relative content of kaolinite without the presence of mica muscovite or albite, but the presence of the mineral phase gibbsite (aluminum hydroxide) is identified. According to the quantitative XRD analysis (Table 3), the gibbsite content is 13 wt%, which together with the kaolinite content of 85 wt%, indicates that the rest of the mineral phases do not exceed 2 wt%. The ICDD card numbers used for peaks indexation in Figure 1 are the following: the K_TA_1 and K_TA_2 samples (Kaolinite: 00-001-0527; Quartz: 01-070-7344; Muscovite Mica: 00-001-1098; Albite:00-009-0466); and the K_CH sample (Kaolinite: 01-089-8538; Quartz: 00-005-0490; Gibbsite: 01-078-1782; Rutile: 01-078-0318).

### 3.2. Obtaining Geopolymer Materials: XRD Study

**Figure 2:**
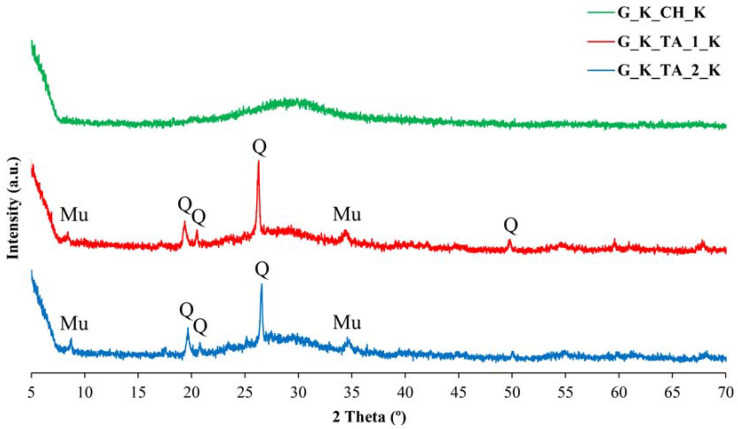
XRD of the obtained geopolymers synthesized with potassium: G_K_CH_K (green line), G_K_TA_1_K (red line), G_K_TA_2_K (blue line). Symbols: Q = Quartz, Mu = Muscovite Mica.

**Figure 3:**
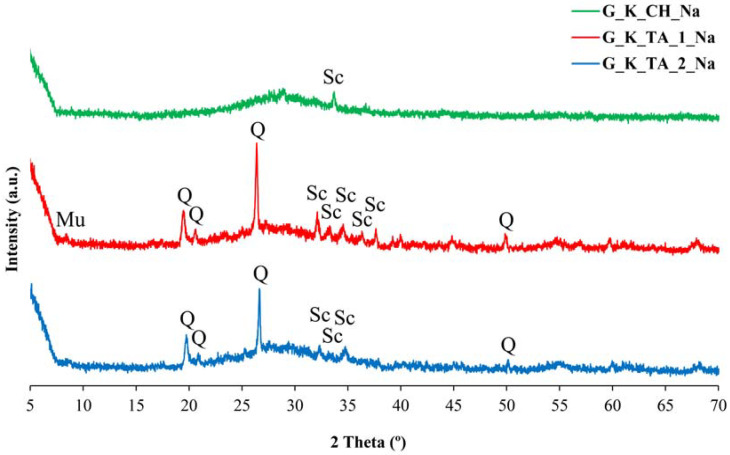
XRD of the obtained geopolymers synthesized with sodium: G_K_CH_Na (green line), G_K_TA_1_Na (red line), G_K_TA_2_Na (blue line). Symbols: Q = Quartz, Mu = Muscovite Mica, Sc = NaHCO_3_ and Na_2_CO_3_.

The XRD diffractograms corresponding to the reaction products between metakaolinite and the basic silicate solutions are presented in Figure 2 and Figure 3.

Reaction of metakaolinite formation [98]:

$${Al}_{2}{Si}_{2}O_{5}{\left(OH\right)}_{4}\overset{400\;{}^{\circ}C-700\;{}^{\circ}C}{\to }{Al}_{2}{Si}_{2}O_{7}+{2H}_{2}O$$

Formation of geopolymers (with Na or K):

$${Al}_{2}{Si}_{2}O_{7}+\left\{\left.\begin{array}{c} \mathrm{Sodium}\;\mathrm{and}\;\mathrm{potassium}\;\mathrm{silicate}\;\mathrm{solutions}\\ \mathrm{alkaline}\;\mathrm{hydroxides}:\;\mathrm{KOH},\;\mathrm{NaOH} \end{array}\right\}\begin{array}{c} Molarratios\\ Si/K=0.58\\ Si/Na=0.56 \end{array}\right.$$

The geopolymers obtained using the Algerian kaolinites K_TA_1 and K_TA_2 show an amorphous character as studied by XRD, but some diffractions corresponding to mineral phases present in the original samples are easily identifiable, although with lower intensities of these diffractions. These mineral phases persist with their crystalline character, as they have not reacted (or have partially reacted) with the alkaline silicates containing KOH (Figure 2) or NaOH (Figure 3). For example, quartz (characteristic peak at 20° of 2θ) designated Q in XRD, as well as muscovite mica (characteristic peak at 35° of 2θ), designated Mu in XRD. According to previous studies [99], the existence of diffraction peaks corresponding to quartz and muscovite mica in the diffractograms of the obtained geopolymers indicates that these two crystalline phases do not participate in the geopolymerization process or reaction, but the diffraction intensities are lower, which is associated with a dilution effect [100]. The ICDD card numbers used for peaks indexation in Figure 2 and Figure 3 are the following: Figure 2 (Quartz: 00-001-0649; Muscovite Mica: 00-002-0464); and Figure 3 (Quartz: 00-002-0471; Muscovite Mica: 00-033-1161).

The geopolymers prepared from kaolin K_CH are characterized by a very broad hump in their diffractogram in the 22–35° 2θ XRD region, which is typical of the diffractograms of amorphous geopolymers [5,9,10,18,37]. Likewise, the diffractograms of the Na-bearing geopolymers obtained from kaolin K_TA_1 and K_TA_2, and even in kaolin K_CH (Figure 3), show peaks of varying intensity at 35°, 33°, 34°, 36° and 37° 2θ that have been identified as belonging to sodium carbonate and/or bicarbonate, more intense in the case of the G_K_TA_1_Na geopolymer. Their presence is associated with the existence of somewhat excessive relative NaOH content after the geopolymerization process, or else it occurred prior to the geopolymerization chemical reaction. The NaOH compound reacts rapidly with atmospheric CO_2_ when exposed to air to form this type of carbonate species. This fact is well known when performing classical volumetric measurements with NaOH titrated solutions that have to be protected from the action of CO_2_ from the air [101].

Previous studies, already mentioned in the introductory part of this paper, on the influence of the starting raw materials in the production of geopolymers have emphasized the nature of the raw materials, kaolin or by-products, some natural and some not, the impurities present and the influence of the heat treatment temperature used for the activation of the raw materials. Some of these studies propose the use of 2:1 clay minerals [10,18,38], but mainly metakaolinite (from kaolinite, 1:1 silicate) is the main precursor in the formation of geopolymer products [5,9,15,36,37,38,80,81,86,91,97]. According to the literature, this is due to the higher reactivity and purity of metakaolinite with respect to other possible raw materials [94], and even the presence of other minerals of the kaolinite group, such as halloysite, has also been found to result in better properties of the obtained geopolymer [9,95]. By effect of the strongly basic alkaline treatment, or “alkaline activation”, the dehydroxylated (metakaolinite) or “thermally activated” product is first hydroxylated and then a polycondensation reaction takes place resulting in the geopolymer as three-dimensional cross-linked chains of polysialates [2,36].

### 3.3. Obtaining Geopolymer Materials: FTIR Study

**Figure 4:**
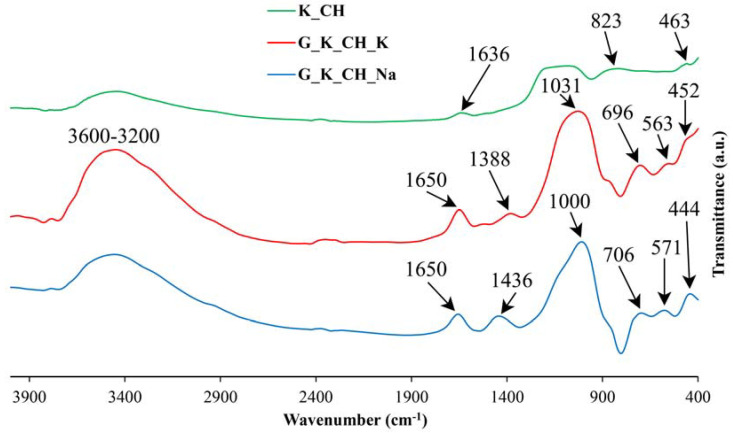
FTIR spectra corresponding to metakaolinite obtained from sample K_CH and the geopolymers obtained with K or Na: K_CH (green line), G_K_CH_K (red line), G_K_CH_Na (blue line).

**Figure 5:**
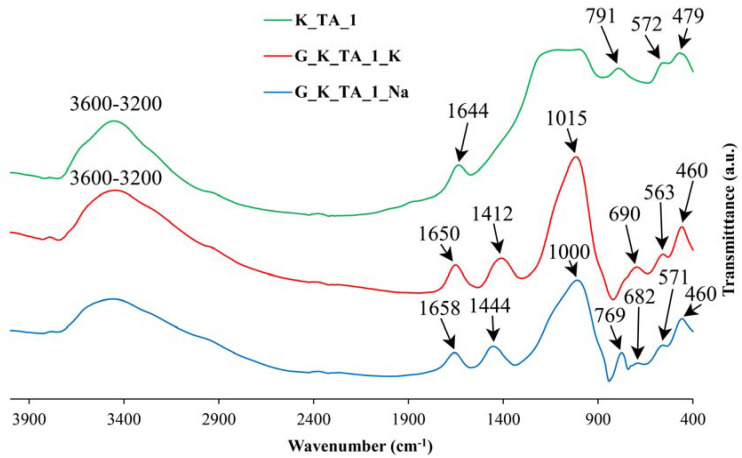
FTIR spectra corresponding to metakaolinite obtained from sample K_TA_1 and the geopolymers obtained with K or Na: K_TA_1 (green line), G_K_TA_1_K (red line), G_K_TA_1_Na (blue line).

**Figure 6:**
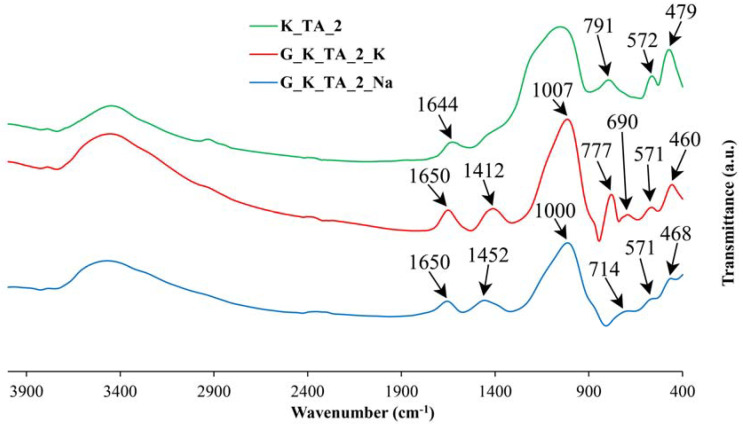
FTIR spectra corresponding to metakaolinite obtained from sample K_TA_2 and the geopolymers obtained with K or Na: K_TA_2 (green line), G_K_TA_2_K (red line), G_K_TA_2_Na (blue line).

As the XRD technique has the limitations inherent to the amorphous or non-crystalline nature of the geopolymers, it is of interest to study the materials obtained by means of a spectroscopic technique such as FTIR. Figure 4, Figure 5 and Figure 6 include the FTIR spectra of calcined kaolins (heat-treated at 750 °C) and the geopolymers obtained, containing Na or K.

The results show that the main bands in the FTIR spectra are those of the calcined kaolin (heat-treated at 750 °C). The results show that the main bands at 1095 cm^−1^, 1079 cm^−1^ and 1134 cm^−1^ in the FTIR spectra of samples K_TA_1, K_TA_2 and K_CH, respectively, are shifted to lower wavenumbers when the geopolymeric materials are obtained (range 1000–1015 cm^−1^), while the lowest shifts are observed in sodium-based formulations compared to potassium-based formulations assigned to T-O stretching bands, where T can be Si and Al, which proves the rearrangement of the lattices and, consequently, the geopolymerization process [38]. In geopolymers, the most intense bands near 444–468 cm^−1^ are assigned to Si-O bending modes. Symmetric Si-O-Si stretching, Al-OH stretching and double ring stretching vibrations have been assigned in the regions 690–714 cm^−1^, 850 cm^−1^ and 563–571 cm^−1^, respectively.

On the other hand, the stretching modes are very sensitive to the Si-Al composition of the lattice and can be shifted to lower frequencies as the number of tetrahedral Al atoms increases [35]. Likewise, in all the samples studied, an increase in the intensity of the bands of the FTIR spectra can be observed in the region between 1600 and 3450 cm^−1^, associated with the bending (H-O-H) and stretching (-OH) vibrations of weakly bound water molecules adsorbed on the surface of both the calcination products (metakaolinite) and the geopolymers or trapped in the cavities of the solid materials. The bands at 1388–1452 cm^−1^ that are also observed in all these spectra are characteristic of M_2_CO_3_ and MHCO_3_ carbonates with M = Na or K [102]. For the case of the geopolymer obtained from K_CH, it can be deduced that the less pronounced changes in this material, appreciated by the FTIR technique, can be associated to a more stable cross-linking structure of the geopolymer network that has been produced in the alkaline chemical activation reaction. In fact, it has already been indicated that the formation of a geopolymer lattice results from solid raw materials containing aluminosilicates modified by “thermal activation” and treated with alkalis in solution, reagents used for their “chemical activation”, which dissolve them; the bonds between Si, Al, K, Na and O are drastically modified and this structural reorganization, when revealed by FTIR, makes it possible to define some domains of existence of the geopolymers obtained [38].

### 3.4. SEM-EDS Microstructural Study of the Geopolymer Materials

**Figure 7:**
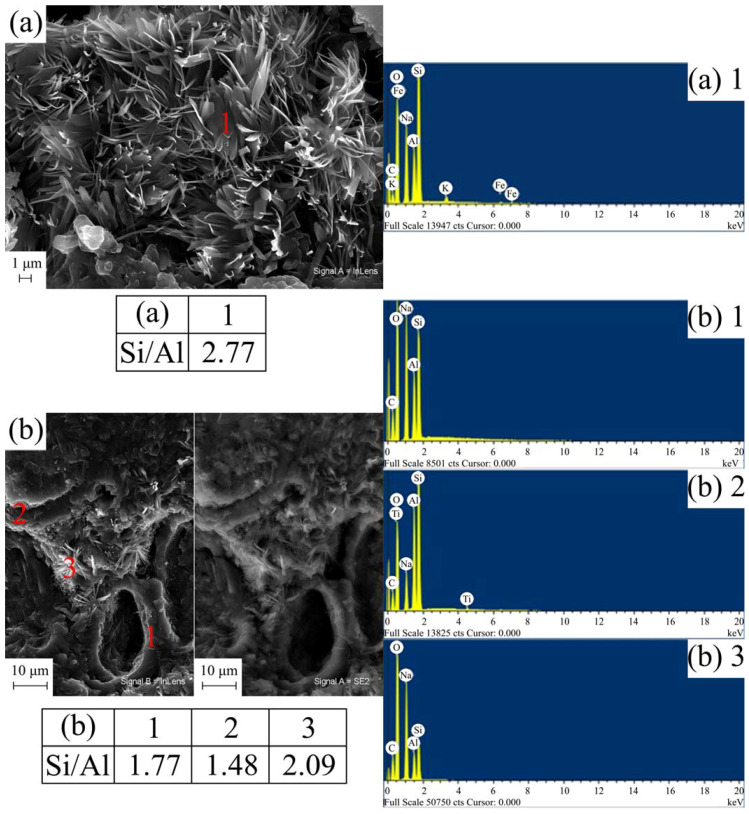
SEM micrographs with EDS analysis in different areas of some geopolymers obtained with the heat-treated kaolins K_TA_2 and K_CH and an alkaline solution containing sodium: (**a**) G_K_TA_2_Na; (**b**) G_K_CH_Na.

**Figure 8:**
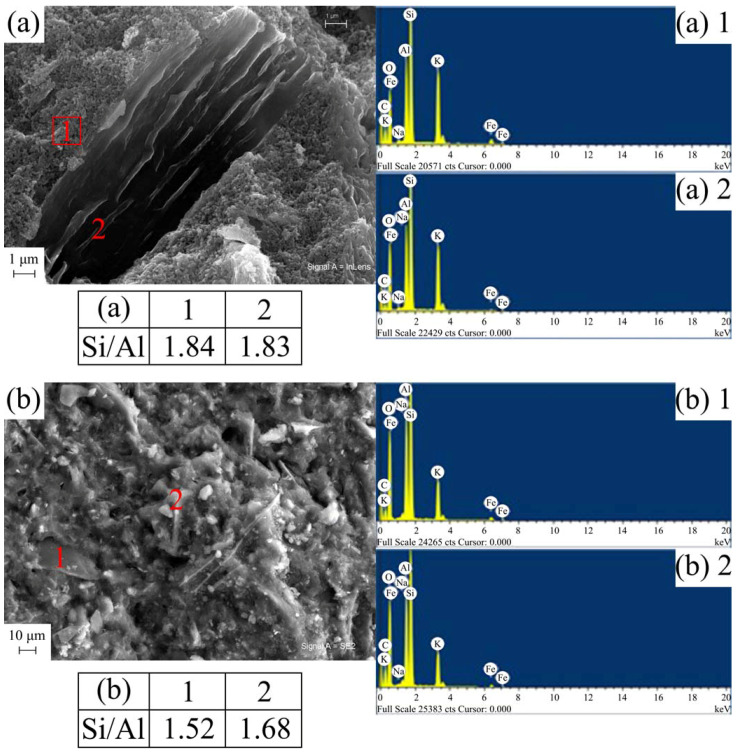
SEM micrographs with EDS analysis in different areas of some geopolymers obtained with the heat-treated kaolins K_TA_2 and K_CH and an alkaline solution containing potassium: (**a**) G_K_TA_2_K; (**b**) G_K_CH_K.

Regarding the SEM microstructural study of the geopolymer materials, complemented by chemical analysis by EDS, Figure 7 and Figure 8 present some of the most outstanding results.

The geopolymer materials based on K_TA_1 show the same microstructure as those based on K_TA_2. Therefore, those corresponding to K_TA_2 and K_CH have been included. The remains of particles with laminar morphology, abundant microcracks and the presence of pores have been found, being the main elements Si, Al, Na and O according to EDS analysis (Figure 7). These pores have been produced in the chemical attack of the metakaolinite lamellae, originated from the thermal treatment of the kaolinite original lamellae, with the basic alkaline solution. In part, they could be associated with the air trapped in the material and the effect of the agitation, which is in agreement with results from the literature [37,94]. The abundance of the microcracks observed can be attributed to the shrinkage that the geopolymer undergoes in the heat treatment process at 70 °C for 24 h, when the water is progressively removed and the polycondensation process that originates the geopolymer takes place.

On the other hand, according to authors such as Davidovits [5], Duxson et al. [12], Khale and Chaudhary [36] and Pimraksa et al. [103], the Si/Al molar ratio significantly affects the degree of polymerization obtained in the geopolymer. The molar ratios of the geopolymers obtained in this study are shown together with the corresponding micrographs. Thus, these values range for the sodium-activated geopolymers in Figure 7, G_K_TA_2_Na and G_K_CH_Na, from 2.77 (geopolymer obtained from K_TA_2) as a maximum to 1.48 as a minimum (geopolymer obtained from K_CH). In the case of Figure 8 (see EDS analysis), the geopolymers obtained with alkaline potassium activator G_K_TA_2_K and G_K_CH_K have somewhat lower molar ratios, ranging from 1.83 (geopolymer obtained from K_TA_2) to 1.52 (geopolymer obtained from K_CH). According to the classification scheme proposed by Davidovits [5], when the Si/Al ratio is 1, the geopolymer is a “sialate, poly(sialate)” (-Si-O-Al-O-); when Si/Al = 2, it is a “sialate-siloxo, poly(sialate-siloxo)” type (-Si-O-Al-O-Si-O-) and when Si/Al = 3, then it is a “sialate-disiloxo, poly(sialate-disiloxo)” type (-Si-O-Al-O-Si-O-Si-O-) and if Si/Al > 3 is satisfied, then it is a “poly(sialate-multisiloxo)” type [104]. According to this criterion, in the present case, taking into account the Si/Al chemical composition ratios, the geopolymers obtained would be more of the sialate-siloxo type.

Finally, the SEM micrographs of Figure 8 correspond to the metakaolinite samples designated K_TA_2 and K_CH, treated with potassium silicate solution containing KOH to obtain geopolymer. The glassy character of this type of geopolymeric sample can be seen in the micrographs, which is in agreement with previous studies already mentioned [37,94] and corresponds to Si/Al ratios = 2–2.5.

## 4. Conclusions

The chemical (Table 2) and mineralogical (Table 3) characterization of three kaolins used in the ceramic industry, K_TA_1, K_TA_2 and K_CH, investigated in this work as raw materials for obtaining geopolymers, has revealed the high relative content of Al and Si. These kaolins have very low relative contents of impurities except goethite and muscovite, which are in kaolins K_TA_1 and K_TA_2 but minimal in kaolin K_CH. The percentage of kaolinite in the samples, deduced by XRD, varies from 57 and 66 wt% in those designated K_TA_1 and K_TA_2 to a maximum of 85 wt% in K_CH. The latter is the sample that does not contain quartz and muscovite mica as the main mineralogical impurities identified by XRD, unlike the previous ones, although it contains gibbsite (13 wt%).

By thermal treatment at 750 °C/1 h of the ground and sieved industrial kaolins (<80 μm), the dehydroxylation of the kaolinite present and the formation of metakaolinite were achieved, with an amorphous structure according to the XRD results, in addition to the persistence of secondary crystalline phases that are not transformed by this treatment. Each of these thermally activated samples K_TA_1, K_TA_2 and K_CH were used as raw materials to obtain geopolymers. By means of a reaction in a strongly basic medium, using aqueous solutions of sodium and potassium silicates containing the respective hydroxides, following a treatment at 70 °C for 24 h, a structural reorganization of the metakaolinite and polycondensation of the product obtained has been produced, which has given rise to the obtaining of geopolymer materials with sodium or potassium. The obtained geopolymers have been characterized by different techniques (XRD, FTIR and SEM-EDS), showing an amorphous character by XRD (Figure 2 and Figure 3) and characteristic bands in the FTIR spectra (Figure 4, Figure 5 and Figure 6).

The examination of the resulting microstructures by SEM-EDS has allowed to observe the presence of microcracks and pores in some Na-containing geopolymer materials when using sodium silicate and NaOH (Figure 7), attributed to the chemical and thermal treatments carried out, which give rise to a shrinkage process when the polycondensation that originates the geopolymers takes place. Likewise, it has been found that if sodium silicate and NaOH are used as basic agents, carbonate species originate, which are evidenced by XRD and FTIR, with a morphology of elongated crystals that are revealed by SEM, and their composition is confirmed by EDS. It has been deduced that there is an effect of atmospheric CO_2_ that induces its formation by chemical reaction.

Finally, taking into account the results of this study in terms of chemical composition, it is concluded that the geopolymeric materials obtained are of the sialate-siloxo type. As future research, several studies on these materials are proposed, including their mechanical properties and their potential applications as coatings for metallic substrates to act as thermal barriers, geopolymer cements useful as refractory adhesive material for metals and gaskets, materials applied to civil engineering, geotechnical engineering or mining engineering, and as replacement materials for cement in the manufacture of foundations or stabilization of clayey soils for road paving, as well as being materials of interest for their resistance to acids.

## Figures and Tables

**Table 1:** Composition of the mixtures prepared according to the molar ratio of species present.

Samples	Si/Al	Si/Na	Si/K	Na/Al	K/Al
G_K_CH_K	1.24	-	1.69	-	0.73
G_K_TA_1_K	1.81	-	1.76	-	1.03
G_K_TA_2_K	1.62	-	1.61	-	1.01
G_K_CH_Na	1.53	1.29	-	1.18	-
G_K_TA_1_Na	2.18	1.13	-	1.92	-
G_K_TA_2_Na	1.98	1.33	-	1.48	-

**Table 2:** Chemical composition (XRF, wt%) of kaolins used as raw materials to obtain geopolymers. LOI: Loss on ignition at 1000 °C.

	K_CH	K_TA_1	K_TA_2
SiO_2_	40.09 ± 0.5	49.30 ± 0.5	48.50 ± 0.5
Al_2_O_3_	42.44 ± 0.3	33.00 ± 0.3	33.90 ± 0.3
Fe_2_O_3_	0.46 ± 0.05	2.37 ± 0.05	2.37 ± 0.05
TiO_2_	0.63 ± 0.05	0.24 ± 0.05	0.21 ± 0.05
CaO	0.18 ± 0.02	0.08 ± 0.02	0.08 ± 0.02
MgO	0.05 ± 0.01	0.40 ± 0.01	0.39 ± 0.01
K_2_O	0.04 ± 0.01	2.92 ± 0.01	2.92 ± 0.01
Na_2_O	0.06 ± 0.01	0.09 ± 0.01	0.08 ± 0.01
LOI	16.00 ± 0.3	10.50 ± 0.3	10.50 ± 0.3

**Table 3:** Mineralogical composition (XRD, wt%) of kaolins used as raw materials to obtain geopolymers.

	K_CH	K_TA_1	K_TA_2
Kaolinite	85 ± 2	57 ± 2	66 ± 2
Quartz	-	13 ± 2	9 ± 2
Muscovite	-	16 ± 2	13 ± 2
Albite	<5	<5	<5
Orthoclase	-	8 ± 2	8 ± 2
Anatase/Rutile	<5	<5	<5
Goethite	-	<5	<5
Gibbsite	13 ± 2	-	-

## Data Availability

Data are contained within the article.
